# Modalities and Determinants of Career Paths in Pediatrics: A Survey of Former Pediatric Residents From Lille University Medical Center

**DOI:** 10.3389/fped.2021.715269

**Published:** 2021-11-22

**Authors:** Charlotte Héritier-Laffargue, Chloé Takvorian, Maeva Kyheng, Sylvie Nguyen, François Dubos, Alain Martinot

**Affiliations:** ^1^CHU Lille, Pediatric Emergency Unit and Infectious Diseases, Lille, France; ^2^CHU Lille, Department of Biostatistics, Lille, France; ^3^Univ. Lille, ULR 2694 METRICS: Evaluation des Technologies de Santé et des Pratiques Médicales, Lille, France; ^4^CHU Lille, Department of Pediatric Neurology, Lille, France

**Keywords:** pediatrician, career path, residency, hospital practice, private practice, satisfaction, job

## Abstract

There is currently a shortage of pediatricians in the Nord-Pas-de-Calais (NPC) area of France. The shortage affects both hospital positions (since many departures are not replaced) and private practice. The objectives of the present study were to (i) describe the career paths of former pediatric residents from Lille University Medical Center, (ii) identify factors associated with leaving NPC and leaving hospital-based practice, and (iii) compare the characteristics associated with the various types of practice.

**Methods:** Pediatric residents having started their residency at Lille University Medical Center between 1993 and 2013 were invited to fill out an online questionnaire. Main outcomes were leaving NPC and leaving hospital practice. The event rate at different times over a 10-year period was determined using the Kaplan-Meier method.

**Results:** The response rate was 92% (284 out of 310 invited respondents): 61% had changed their place or type of practice at least once, 54% had moved to a different city, and 41% had left NPC. Having trained elsewhere than in Lille and the lack of a chief assistant specialist position in NPC were independently associated with leaving NPC. 73% of the respondents were currently in hospital-based practice. Having started residency after 2003, taking a sabbatical during the residency and not training in a subspecialty (other than general pediatrics) were independently associated with leaving hospital-based practice. The stated reasons for leaving hospital-based practice were on-call duties (according to 71% of the respondents), overwork (46%), family reasons (34%), and a poor atmosphere at work (34%). Hospital-based pediatricians were more active in research and teaching. They worked an average of 13 h more per week than the other respondents, and were less satisfied with their choice of professional activity and their work-life balance.

**Conclusion:** Changes in the place or type of practice have become frequent. With the recent resurgence of interest in private practice, leaving hospital is reportedly associated with better working conditions, greater satisfaction, and a better work-life balance.

## Introduction

From 1984 to 1995, the French health authorities' attention was drawn to the danger of a decline in the number of practicing pediatricians. The number of medical students following a specialist track in pediatrics fell from 250 to 110 (1). This number rose very gradually to 177/year over the next 20 years before ultimately rising sharply to 330. However, this late increase did not cover the population's need for an effective policy on child and adolescent health (2). In 2018, there were 8,205 practicing pediatricians in France, i.e., 67 per 100,000 children under the age of 15 (52 per 100,000 in the former Nord Pas de Calais (NPC) region) (3, 4). Sixty-eight percent of the pediatricians in France (78% in NPC) were salaried employees (in hospitals, mainly), 22% (13% in NPC) were in private practice, and 11% (9% in NPC) combined the two activities (5). The medical training curriculum in France is presented and compared with two main English-speaking countries in Supplementary Table 1. The ranking in a national qualifying examination determines the choice of specialty and university town for the residents. Pediatrics was ranked about 20th out of 30 specialties with no significant variation in recent years.

Between 1993 and 2003, Lille University Medical Center (LUMC, the only university medical center in NPC, a region with four million inhabitants, including one million children) offered an average of 12 pediatric residency positions per year. To make up for the regional shortage of pediatricians, this number has gradually increased to between 20 and 23 positions per year since 2011. In contrast, the number of 2-year chief assistant specialist positions at LUMC (i.e., specialist registrars) has not changed—meaning that only 20% of the pediatrics residents (rather than 40% previously) have a chance of obtaining a position as a chief assistant specialist at LUMC. The availability of chief assistant specialist positions in general hospitals increased access to a post-residency job in NPC but did not enable people to train in a subspecialty of pediatrics. In order to increase opportunities for subspecialty training in NPC from the early 2000s onwards, the NPC Regional Health Agency created a number of positions in which a chief assistant specialist worked part-time at LUMC and part-time in a general hospital. This system was extended to the whole country in 2010. NPC had no other specificity in terms of pediatric residency programs and medical training curriculum.

Despite the increase in the number of pediatric residents per year and greater opportunities for employment as a chief assistant specialist, many consultant positions are currently vacant in the NPC region's hospitals. This might be due particularly to early resignation by hospital pediatricians just a few years after their appointment. Pediatricians' careers are becoming more complex, with more frequent moves (from one city or region to another) and changes in the type of practice (with a recent resurgence in the attractiveness of private practice).

We therefore thought it would be interesting to describe these career pathways and to look for their determinants. To this end, we focused on two types of change: leaving the NPC region and leaving hospital-based practice. The main objective of the present survey was to describe the career paths of physicians who had started their pediatric residency at LUMC between 1993 and 2013. The secondary objectives were to identify factors associated with leaving NPC or leaving hospital-based practice, or associated with the various modes of practice.

## Methods

We performed an online, descriptive, observational survey between December 30th, 2019, and March 7th, 2020. All physicians having started a residency in pediatrics at LUMC between 1993 and 2013 (i.e., ending their 4 year residency in 2017 at the latest) were eligible for inclusion. We excluded residents who took the national qualifying examination again with a view to going to another university medical center or changing to a specialty other than pediatrics, and those who had died. The names of the former residents were provided by the Regional Health Agency in the Hauts-de-France region (of which NPC is now part). We identified prospective participants by checking professional directories, the Internet, and personal networks. The participants' e-mail addresses were thus obtained directly or after a phone call. The link to the online questionnaire was sent by e-mail. In the absence of a reply, up to four e-mail and/or phone reminders were sent out.

The five-part, 30-item questionnaire was built and managed with the Google Forms web application (https://docs.google.com/forms). The first part concerned the respondents' characteristics: gender, age, number of children, initial medical education, and criteria for choosing the pediatric residency program at LUMC. The second part concerned the residency period: the various departments' specialties, completion of at least one 6-month internship outside NPC, university degrees in subspecialties, Master's degrees, sabbaticals lasting at least 6 months, burn-out/depression/time off work for psychological reasons, and number of children at the end of the residency. The third part asked the participants about the first 2 years following their residency: whether or not they had become a chief assistant specialist, the type of chief assistant specialist position, qualification in a subspecialty, city and type of practice. The fourth part concerned the rest of the pediatrician's career up until the present time: the number of changes in the place or mode of practice, and the associated reasons. The final section focused on the pediatrician's current practice: the place and mode of practice, the working hours, the number of on-call duties, personal income, and involvement in research, teaching, learned societies or subspecialist groups. Job satisfaction was rated on a Likert scale. Lastly, the participant was asked about any periods of burn-out, depression or other time off work since the end of the residency.

The answers were exported to an Excel® spreadsheet (version 2016, Microsoft Corp., Redmond, WA) and anonymized. The study was registered with the French National Data Protection Commission (*Commission nationale de l'informatique et des libertés* (Paris, France); reference: ID417), and validated by the Ethical Committee of the University Hospital of Lille, attesting that the study got an approval with waiver of informed consent in agreement with French regulations concerning such observational studies.

Two subgroups of residency periods were defined: those starting between 1993 and 2002 and those starting between 2003 and 2013. The 2003 year group was the first to have access to joint LUMC/general hospital chief assistant specialist positions. Subspecialties were defined as specializations in a subspecialty of pediatrics other than general pediatrics. The current place of practice was classified as (i) NPC, (ii) the “home” region (defined as the region where the physician had trained) outside NPC or (iii) another region outside NPC. The type of practice was classified as “hospital-based” (if at least 80% of the working time was spent in a general or university hospital), “non-hospital-based” (if at least 80% of the working time was spent in private or community practice), “mixed” (both hospital-based and non-hospital-based), or “other.”

Categorical variables were expressed as the frequency (percentage). Quantitative variables were expressed as the mean ± standard deviation (SD) or (for a non-normal distribution) the median [interquartile range (IQR)]. The normality of the data distribution was assessed graphically and by using the Shapiro-Wilk test. Respondents were compared with non- respondents in a chi-squared test or Fisher's exact test. The event rate at different times over a 10-year period for various groups (according to the place where they had trained, chief residency in NPC or not, and the year group) was determined using the Kaplan-Meier method. In a univariate analysis, factors associated with practicing in NPC and practicing in a hospital 10 years after residency were probed in a log rank test (for qualitative variables) or a Cox model (for quantitative variables). Factors associated with a *p* < 0.20 in these analyses were fed into a multivariable Cox model with backward stepwise selection. Log-linearity assumptions were tested for the quantitative variables and the proportional hazards assumption was tested for all variables. Factors associated with current practice (hospital vs. non-hospital) were analyzed using a Cochran-Armitage trend test (for ordinal variables), Student's test (for continuous variables) or a chi-squared test (for qualitative variables). All tests were two-tailed, and the threshold for statistical significance was set to *p* < 0.05. The statistical analyses were performed using SAS software (version 9.4, SAS Institute Inc., Cary, NC).

## Results

**Figure 1:**
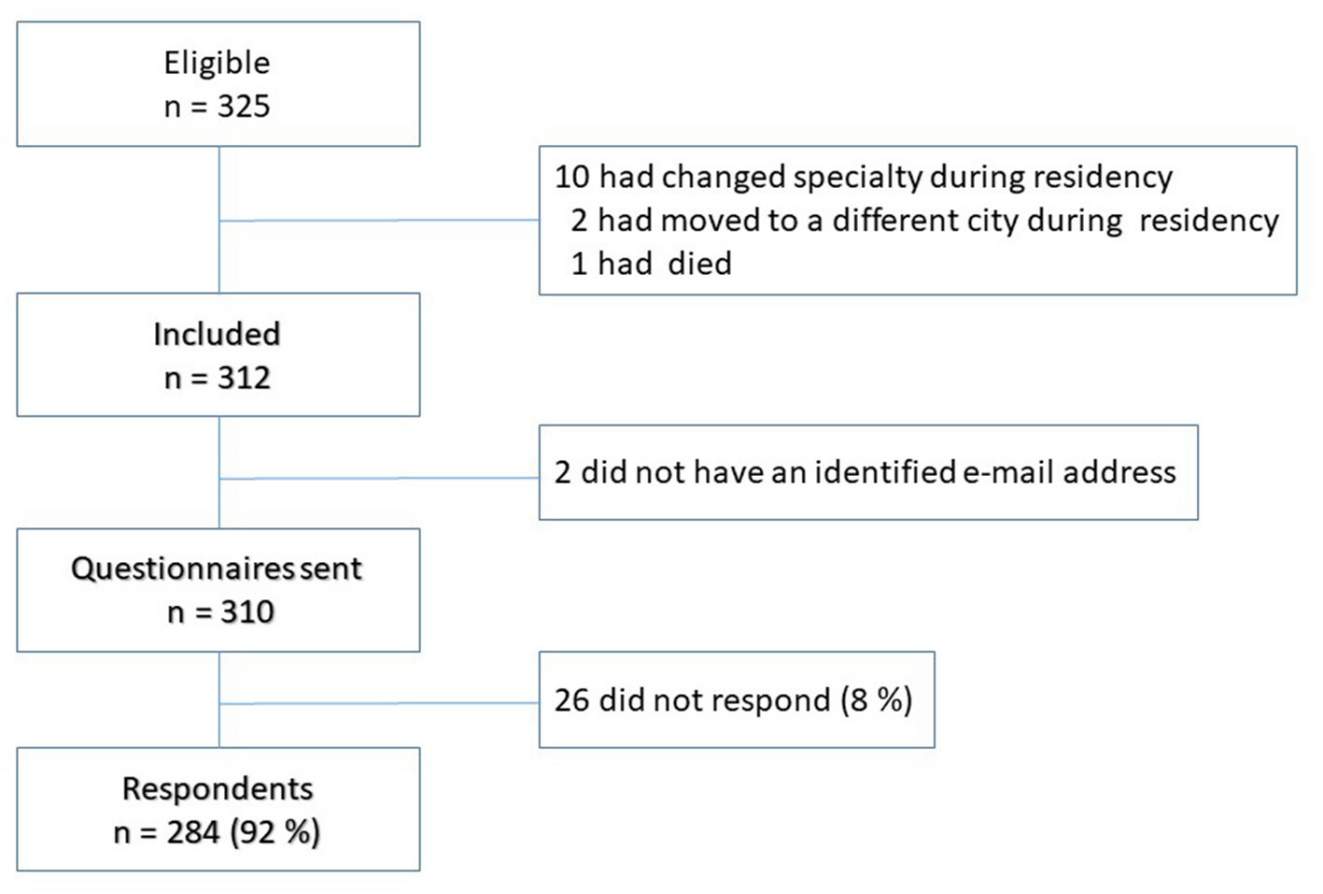
Flow chart of study participants.

A total of 325 residents were eligible for inclusion. We excluded 13 residents for reasons shown in Figure 1, and 2 others for whom we could not find a valid e-mail address. 284 (92%) of the 310 residents with a valid e-mail address replied to the survey. The 26 non-respondents did not differ from the 284 respondents in terms of gender, residency period, current practice in NPC, and type of practice.

Two hundred and fifty of the respondents (88%) were women. The respondents had trained in Lille (38%), Paris (24%), another French city (35%), or outside France (2%). LUMC was the first choice for 76% of the residents (98% of Lille graduates and 62% of those having studied elsewhere; *p* <10^−4^). The main reason given for eventually choosing LUMC by those for whom it was not their first choice was a ranking not sufficiently good for a pediatrics assignment in their first choice city (94%). Thirty six percentage had had at least one 6-month sabbatical during their residency (Table 1): this was mainly due to working as a locum in private practice (50%), the need for a break from work (35%), the arrival of a child (26%), or travel/humanitarian missions (20%). Ninety percentage reported that they had trained for a pediatric subspecialty (Table 1).

**Table 1:** Characteristics of the residency and the first 2 years post-residency for former pediatric residents at Lille University Medical Center (*n* = 284).

**Residency**	***n***	**%**
Inter-UH residency (at least one 6-month period outside NPC)	52	**18**
Sabbatical (at least one 6-month period)	102	**36**
Master's degree	74	**26**
University or inter-university degree (at least one)	272	**96**
Burn-out/depression during residency	34	**12**
Time off work for psychological reasons during residency	6	**2**
Parenthood by the end of the residency	87	**31**
Specialties reported^*^		
Neonatology and/or pediatrics in a maternity hospital	72	**25**
General pediatrics	50	**18**
Pulmonology and/or allergology	35	**12**
Hepatogastroenterology	30	**11**
Neurology	27	**10**
Endocrinology and/or diabetology	27	**10**
Emergency medicine	22	**8**
Hematology	15	**5**
Critical care medicine	13	**5**
Oncology	12	**4**
Cardiology	10	**4**
**Position/status after residency**		
Chief assistant specialist:		
in a university hospital with university duties	94	**33**
in a university hospital with no university duties	17	**6**
full-time in a general hospital	43	**15**
on a time-sharing basis (university and general hospital)	65	**23**
Attending physician or contract pediatrician in a general hospital	30	**10**
Attending physician or contract pediatrician in a university hospital	15	**5**
Private practice	14	**5**
Community practice	3	**1**
University research (Ph.D. etc.)	3	**1**
**Chief assistant specialist positions (*****n*** **=** **219)**		
Chief assistant specialist positions in NPC	165	**75**
Chief assistant specialist positions that were reportedly of value for the current activity	197^**^	**99**
Attending physician in the same hospital at the end of the chief assistant specialist position	135^***^	**66**

* *Respondents could report more than one specialty, if applicable. The other reported specialties concerned <4% of the respondents: infectious diseases (n = 9), nephrology (7), rheumatology and/or internal medicine (5), social pediatrics (4), palliative care (4), metabolic diseases (3), medical genetics (2), pain (1), dermatology (1), child psychiatry (1), or others (hypnosis/homeopathy) (1)*.

** *missing data n = 19*.

*** *missing data n = 14*.

*UH, university hospital; NPC, Nord Pas-de-Calais region. Bold values are represented as percentages*.

Seventy seven percentage of the respondents had been a chief assistant specialist (Table 1). Sixty one percentage of the pediatricians had changed their type and/or place of practice at least once. The median [IQR] number of changes was 1 [0–2], and the median time interval between the end of the residency and the first change was 2 years. The stated reasons for each type of change are detailed in Table 2. The main reason for changing from practice in a general hospital to private practice was the on-call activity (71%).

**Table 2:** The main reasons stated for each major type of career change for former pediatric residents at Lille University Medical Center (changing city without changing the type of practice, and the various changes in the type of practice).

	**Change in city only**	**From UH to GH**	**From GH to PP**	**From UH to PP**	**From GH to UH**
	***n* = 117 (38%)**	***n* = 42 (14%)**	***n* = 41 (13%)**	***n* = 22 (7%)**	***n* = 18 (6%)**
Family reasons, *n* **(%)**	56 **(48)**	11 **(26)**	14 **(34)**	10 **(45)**	7 **(39)**
Work atmosphere, *n* **(%)**	20 **(17)**	10 **(24)**	14 **(34)**	5 **(23)**	5 **(28)**
No positions available, *n* **(%)**	18 **(15)**	15 **(36)**	3 **(7)**	3 **(14)**	2 **(11)**
On-call activity, *n* **(%)**	11 **(9)**	2 **(5)**	29 **(71)**	6 **(27)**	2 **(11)**
Stress/burnout, *n* **(%)**	13 **(11)**	7 **(17)**	19 **(46)**	3 **(14)**	1 **(6)**
Working hours, *n* **(%)**	11 **(9)**	7 **(17)**	11 **(27)**	5 **(23)**	1 **(6)**
Salary, *n* **(%)**	2 **(2)**	3 **(7)**	4 **(10)**	2 **(9)**	0 **(0)**
Type of activity, *n* **(%)**	10 **(9)**	6 **(14)**	6 **(15)**	3 **(14)**	6 **(33)**
Inadequate resources, *n* (**%)**	4 **(3)**	0 **(0)**	3 **(7)**	1 **(5)**	2 **(11)**
Job opportunity, *n* **(%)**	10 **(9)**	3 **(7)**	1 **(2)**	0 **(0)**	2 **(11)**
Change in subspecialty, *n* (**%)**	2 **(2)**	0 **(0)**	1 **(2)**	0 **(0)**	0 **(0)**
Personal reasons/choice, *n* **(%)**	16 **(14)**	3 **(7)**	3 **(7)**	3 **(14)**	0 **(0)**
University (fellowship), *n* (**%)**	8 **(7)**	0 **(0)**	0 **(0)**	0 **(0)**	1 **(6)**

*UH, university hospital; GH, general hospital; PP, private practice*.

*The two most frequent reasons for each type of change are highlighted in gray*.

*Other types of less frequent changes: change in type of activity (6%), change in establishment while maintaining the same type of activity (6%), UH to community pediatrics (5%), PP/community to GH/UH (3%), PP/community to community only (2%). Bold values are represented as percentages*.

Seventy three percentage of the respondents were currently working primarily in a hospital (Table 3). Former LUMC residents were working in all of the 18 hospitals in NPC region that had at least one pediatrics department. Fifty eight percentage of the respondents and 83% of the pediatricians in private practice were working <20 km from LUMC. The great majority of the pediatricians somewhat or fully endorsed their choice of pediatrics (87%) or would recommend it to a future resident (73%). Thirty percentage somewhat or fully agreed that they did not have a satisfactory work-life balance.

**Table 3:** Characteristics of the current practice of former pediatric residents at LUMC (*n* = 284).

Age, years, mean ± standard deviation (range)	39.3 ± 6.5	(29–55)
Time since completion of residency, years, mean ± standard deviation (range)	10.8 ± 6.2	(0.5–23)
At least 1 child, *n*, %	220	**77**
At least 3 children, *n*, %	82	**29**
Main type of practice^*^, *n*, %		
Hospital-based^*^	206	**73**
Non-hospital-based^*^ (private practice, community, or non-hospital-based salaried employment)	73	**26**
Mixed^*^ or other	5	**2**
**Type of practice (giving more than one answer was possible, for any type of practice—even part-time), *n*, %**		
General hospital	130	**46**
University hospital	102	**36**
Private practice in a group practice	33	**12**
Community practice (schools, preventive medicine, etc.)	26	**9**
Private practice in cabinet alone	21	**7**
Maternity hospital	19	**7**
Private practice in a private hospital	7	**2**
Other places	8	**3**
Monthly income class^**^, euros, *n*, %		
<3,000	37	**13**
3,000–4,500	133	**47**
4,500–6,000	87	**31**
>6,000	22	**8**
Research activities, *n*, %	119	**42**
Teaching activities, *n*, %	137	**48**
Full-time work, *n*, %	197	**69**
Weekly working hour, median [IQR]	50	[45–60]
Burnout/depression during the career, *n*, %	59^***^	**21**
Time off work for psychologic reasons during the career, *n*, %.	28^***^	**10**
At least 1 hospital-based on-call duty per month, *n*, %.	158	**56**
At least 1 hospital-/home-based on-call duty per month, *n*, %.	45	**16**
At least 1 home-based on-call duty per month, *n*, %.	94	**33**
No on-call duties, *n*, %.	71	**25**

* *Defined as “hospital-based” if at least 80% of the working time was spent in hospital, “non-hospital-based” if at least 80% of the working time was spent in private or community practice or outside hospital, and “mixed” for the remaining possibilities*.

** *5 did not wish to answer*.

*** *missing data n = 6. Bold values are represented as percentages*.

### Leaving the NPC Area

Although 59% of the respondents were still working in NPC over 10 years after residency, 15% had returned to the region where they had trained and 26% had gone to a region other than where they had trained. The factors independently associated with migration from NPC were training outside and the absence of a chief assistant specialist position in NPC (Table 4 and Supplementary Figures 1, 2). Starting residency in 1993–2002 was associated with a greater likelihood of leaving NPC in the univariate analysis only (Table 4).

**Table 4:** Univariate and multivariate analyses of factors associated with leaving the Nord Pas-de-Calais (NPC) area within 10 years of the end of the residency.

	**Leaving NPC after 2 yrs**	**Leaving NPC after 5 yrs**	**Leaving NPC after 10 yrs**	*p* ^*^	**aHR [95%CI]**	*p* ^**^
**Start of residency**				** <0.001**	^#^	
1993–2002	36 (33)	51 (47)	61 (56)			
2003–2013	39 (22)	49 (29)	52 (32)			
**Chief assistant specialist in NPC**				** <0.001**		** <0.001**
No	69 (58)	76 (64)	78 (67)		1.00	
Yes	6 (4)	24 (16)	35 (27)		0.25 [0.17–0.38]	
**Initial medical education**				** <0.001**		** <0.001**
Lille	13 (12)	16 (15)	17 (17)		1.00	
Outside Lille but Lille 1st choice	36 (32)	50 (46)	59 (60)		3.44 [2.00–5.93]	
Outside Lille, Lille not 1st choice	26 (41)	34 (55)	37 (61)			
Inter-hospital residency^***^				0.26	^§^	
No	59 (25)	78 (35)	89 (42)		3.33 [1.86–5.98]	
Yes	16 (31)	22 (43)	24 (50)			
**Master's degree**				0.51	^§^	
No	54 (26)	73 (36)	85 (46)			
Yes	21 (28)	27 (37)	28 (39)			

*Values are expressed as number (percentage)*.

*aHR, adjusted Hazard Ratio; CI, confidence interval; NPC, Nord Pas-de-Calais area*.

* *p-values for the univariate analysis obtained using log-rank test;*

** *p-values for the multivariate analysis: Model obtained after backward stepwise selection in cox model including all factors with a p < 0.20*.

# *variables withdrawn from the model after multivariate analysis*.

§ *variables not introduced in the multivariate model*.

*** *at least one 6-month internship in a university hospital outside the NPC region. Bold values p < 0.05*.

### Stopping Hospital-Based Practice

The proportion of pediatricians practicing in a hospital fell steadily over time; 25% of the pediatricians had left hospital-based work 9 years after the end of their residency period. The factors independently associated with leaving hospital-based work were starting residency in 2003–2013 (Table 5 and Supplementary Figure 3) and taking a sabbatical during the residency. In contrast, qualifying in a subspecialty other than general pediatrics was associated with staying in hospital-based position (Table 5).

**Table 5:** Univariate and multivariate analyses of factors associated with leaving hospital within 10 years of the end of the residency.

	**Leaving hospital after 2 yrs**	**Leaving hospital after 5 yrs**	**Leaving hospital after 10 yrs**	*p* ^*^	**aHR [95% CI]**	*p* ^**^
**Start of residency**				0.06		**0.037**
1993–2002	5 (5)	14 (13)	23 (21)		1	
2003–2013	15 (9)	26 (17)	38 (35)		1.80 [1.0–3.11]	
**Chief assistant specialist in NPC**				0.12	^#^	
No	16 (13)	23 (20)	31 (30)			
Yes	4 (2)	17 (12)	30 (25)			
**Gender**				0.42	^§^	
Male	3 (9)	7 (21)	10 (32)			
Female	17 (7)	33 (15)	51 (26)			
**Master's degree**				**0.007**	^#^	
No	18 (9)	33 (17)	52 (32)			
Yes	2 (3)	7 (10)	9 (14)			
**Sabbatical during residency**				**0.006**		**0.009**
No	5 (3)	21 (13)	33 (22)		1	
Yes	15 (15)	19 (20)	28 (38)		2.00 [1.19–3.34]	
**Burnout/depression during residency**				0.42	^§^	
No	16 (6)	36 (15)	57 (28)			
Yes	4 (12)	4 (12)	4 (12)			
**Subspecialty**				** <0.001**		** <0.001**
No	14 (50)	20 (77)	21 (82)		1	
Yes	6 (2)	20 (8)	40 (21)		0.09 [0.05–0.16]	

*Values are expressed as number (percentage)*.

*aHR, adjusted Hazard Ratio; CI, confidence interval; NPC, Nord Pas-de-Calais area*.

* *p-values for the univariate analysis obtained using log-rank test;*

** *p-values for the multivariate analysis: model obtained after backward stepwise selection in cox model including all factors with a p < 0.20*.

# *variables withdrawn from the model after multivariate analysis*.

§ *variables not introduced in the multivariate model. Bold values p < 0.05*.

### A Comparison of Hospital-Based Practice and Private Practice

**Figure 2:**
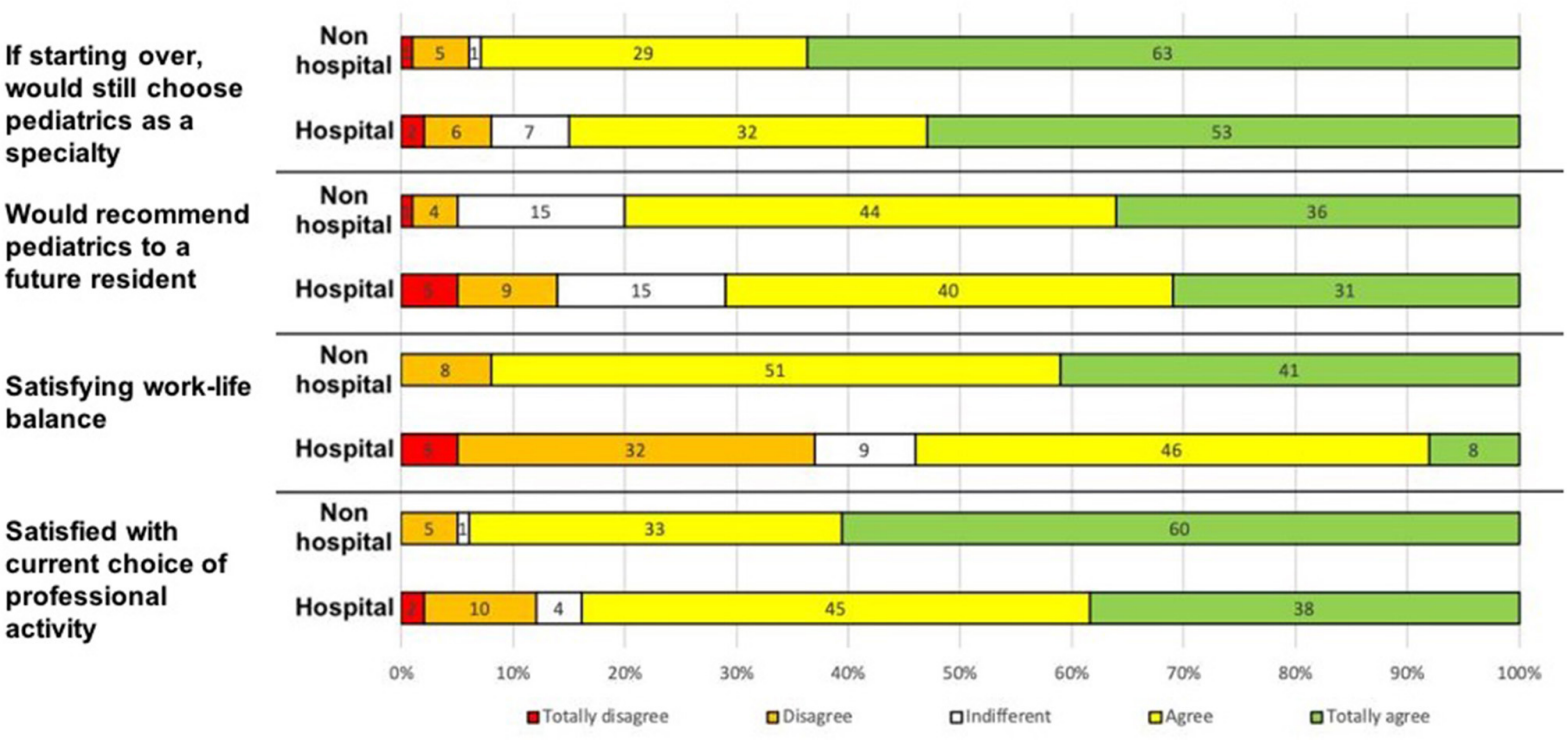
Job satisfaction as a function of the current mode of practice (scored on a Likert scale).

Pediatricians in hospital practice were more likely to be involved in research (48%, vs. 25% of the other respondents, *p* < 0.001) and teaching (54% vs. 33%, respectively; *p* < 0.003) and had a longer mean working week (55 ± 9 h, vs. 42 ± 9 h for the other respondents; *p* < 0.001). Relative to hospital-based pediatricians, pediatricians in non-hospital practice were more likely to report a satisfactory work-life balance and were more often satisfied with their chosen activity (Figure 2).

## Discussion

The results of the present survey highlighted an evolution in the career paths of former pediatric residents in NPC, with frequent changes in the type or place of practice. In particular, the region where the residents had trained was important for the long-term maintenance of their practice in NPC, along with access to a broad range of chief assistant specialist positions (made possible by the development of joint LUMC-general hospital positions). Our results also confirm the renewed attractiveness of private practice, with two types of profiles: less specialized practitioners, who worked as locums in private practice during their residency and settled quickly, and specialized practitioners who settled only after several years of working as hospital practitioners (due to the arduousness of the work, which was mainly linked to frequent on-call duties). Although the great majority of the respondents were satisfied with their choice of pediatrics, they nevertheless flagged up difficulties in combining professional life and family life. These difficulties were more frequently mentioned by hospital pediatricians than by private practitioners, and steered practitioners toward better-defined working hours and fewer on-call duties.

Women accounted for 88% of our respondents. This proportion was 82% in a survey of pediatric residents in western France between 1990 and 2000 (6). The proportion of women is increasing in the medical professions in general and in pediatrics in particular; in January 2018, women accounted for 70% of the pediatricians in France (4). 18% of the residents in NPC and 32% of the residents in western France had carried out an internship in another university hospital (6). Although the proportion of NPC residents having become a chief assistant specialist (77%) was similar to that reported in western France (75%), the status differed: the proportion of university hospital positions was 43% in NPC vs. 84% in western France (6). This is probably due to the low chief assistant specialist/resident ratio at LUMC. The chief assistant specialist position was located in the same region as the residency in 75% of cases in NPC and in 62% of cases in western France. People having been a chief assistant specialist in NPC were less likely to leave the region thereafter (Table 4). In a univariate analysis, respondents having started their residency after 2003 were less likely to leave NPC; however, this factor was no longer statistically significant in a multivariate analysis—probably because post-2002 residents were more likely to have trained in Lille (Table 4). The creation of joint LUMC/general hospital positions increased the availability of chief assistant specialist positions in NPC at a time when the number of residents per year group had increased sharply. These joint LUMC/general hospital positions enable the pediatrician to continue his/her training in a subspecialty at the university hospital while working in a general hospital; these positions are very popular and are sometimes even preferred to university hospital chief assistant specialist positions.

Almost three out of four pediatricians trained in Lille (like those trained in western France) were currently working full-time in a hospital (69 and 74%, respectively) in the region where they had graduated (58 and 59%, respectively) (6). The factors significantly associated with leaving hospital-based practice were residency after 2002, a sabbatical during the residency, and the absence of a subspecialty (other than general pediatrics) (Table 5). At least one subspecialty was declared by 82% of our pediatricians. There was a significant increase in subspecialty training from 59 to 67 % among Canadian pediatric residents between 2004–2007 and 2008–2010 (7).

The renewed interest in private practice has not been observed in other specialties. In dermatology, an increasing interest was observed for salaried activities or mixed (salaried plus private practice) activities 5 to 7 years after the end of residency (8). In radiology, young women stated that maternity might influence their career choices and willed to work in salaried positions to acquire a secure job, even though compensation might be lower (9). Since the French government reformed the medical education system in 2017, the postgraduate diploma in pediatrics has been extended from 4 to 5 years. Furthermore, the number of outpatient and community pediatrics internships has been increased, in order to provide better training in non-hospital-based practice. These outpatient internships did not start in NPC until May 2020 (10, 11). In NPC, 83% of the private practices in pediatrics are located in and around Lille; this prevents children in other parts of the region from being monitored by a pediatrician. Although hospital-based pediatricians and private pediatricians have different skills, missions, and areas of practice, their complementarity is a true asset for the health of infants, older children, and adolescents (2). Transferring pediatricians from one mode of practice to another will not therefore resolve the shortage of pediatricians in France.

Like the former pediatric residents in western France (6), 86% of the former pediatric residents at LUMC were satisfied or very satisfied with their current professional activity and 64% somewhat agreed or strongly agreed that they had a satisfactory work-life balance. Poor work-life balance and intense on-call commitments were the two most common reservations about choosing pediatrics cited by 1st year pediatric specialty trainees in UK (12). A current appropriate work-life balance was reported by 43% among young pediatricians in the USA, for whom three factors (good health, support from colleagues, and sufficient resources for care) were independently associated with greater professional satisfaction, greater personal satisfaction, and a lower prevalence of burnout (13). Others US studies reported that both pediatricians pursuing or not pursuing fellowship training can achieve overall life and career satisfaction (14), and that a large majority of pediatric subspecialists found initial jobs matching their goals for professional responsibilities and clinical care (15).

Episodes of burnout/depression during residency or at some point in the career was mentioned respectively by 12 and 21% of the former residents at LUMC. Ten percent had already taken time off work for psychological reasons. The prevalence of burnout/work exhaustion syndrome and its consequences (depression, suicide, addictive behavior, and job dissatisfaction) may be underestimated in France. These problems reportedly affected 40 to 75% of pediatric residents in the USA (16, 17). In a longitudinal follow-up study, 20 to 35% of pediatricians in the USA reported that they were experiencing burn-out and 58% reported at least one episode within the previous 5 years (18).

The very high response rate to the survey (92%) strongly expressed the interest of physicians to this analysis. The study had several limitations. Firstly, the study's regional scope prevented extrapolation of the results to France as a whole and to other countries. Secondly, the self-reporting of sometimes old data was subject to memory bias. Thirdly, the long (21-year) study period meant that the time interval between the end of residency and the survey date varied markedly (from 2 to 23 years).

Even though the NPC has the second largest quota of pediatric residents in France (after the Ile-de-France region, i.e., Paris area), its pediatrician density is below the national average. The growth in the number of pediatric residents must be maintained regionally and nationally. The greater proportion of residents having trained in NPC and the increase in the number of available chief assistant specialist positions in the region since the early 2000s has made it possible to retain a larger number of LUMC residents. The revived interest in private practice in pediatrics should be beneficial for children in the NPC region but must not endanger the hospital departments' ability to function. A recent review of the literature exploring specific strategies used to enhance recruitment and retention in pediatrics identified six main themes: early advocacy of pediatrics, with early exposure to pediatrics at an undergraduate level, work force diversity and expansion, with use of other healthcare professionals, proactive mentorship, improving working conditions, sustainable career flexibility, and enhancing educational opportunities (19).

## Conclusion

The difficulties of hospital-based practice (and particularly the burden of on-call activity) expressed by our respondents must be reduced by setting up sufficiently large hospital teams (at least seven practitioners for an on-call rota); this might be achieved by bringing together teams from several sites, particularly for on-call duties. A policy to improve the practitioners' working conditions (debriefing sessions, psychological follow-up and support, etc.) should be developed. Access to part-time hospital positions might encourage mixed hospital practice/private practice activities and perhaps attract pediatricians to areas with a shortage of these professionals.

## Data Availability Statement

The raw data supporting the conclusions of this article will be made available by the authors, without undue reservation.

## Ethics Statement

Ethical review and approval was not required for the study on human participants, in accordance with the local legislation and institutional requirements.

## Author Contributions

CH-L, CT, FD, and AM: conception of the study. FD and AM: project administration. CH-L, CT, MK, FD, and AM: methodology. CH-L and CT: investigation. CH-L, CT, MK, and AM: formal analysis. CH-L and AM: drafting of manuscript. CT, MK, SN, FD, and AM: revision of manuscript. All authors contributed to the article and approved the submitted version.

## Conflict of Interest

The authors declare that the research was conducted in the absence of any commercial or financial relationships that could be construed as a potential conflict of interest. The handling editor declared a past co-authorship with one of the authors FD.

## Publisher's Note

All claims expressed in this article are solely those of the authors and do not necessarily represent those of their affiliated organizations, or those of the publisher, the editors and the reviewers. Any product that may be evaluated in this article, or claim that may be made by its manufacturer, is not guaranteed or endorsed by the publisher.
