# Supplementary material for: Modalities and Determinants of Career Paths in Pediatrics: A Survey of Former Pediatric Residents From Lille University Medical Center
Source: Front Pediatr. 2021 Nov 22;9:715269. doi: 10.3389/fped.2021.715269 (PMC8645605; doi:10.3389/fped.2021.715269)
Supplement: Supplementary file 1 [file Table_1.docx]

**Supplementary Table 1.** A comparison of the medical training curricula for pediatrics in France, the UK and the USA. Year 1 is the first year of residency.

| **Years** | **France** | **UK** | **USA** |
| --- | --- | --- | --- |
| Year -6 | / | / | “Pre-med” courses |
| Year -5 | First year of health studies | / | “Pre-med” courses |
| Year -4 | General Diploma in Medical Science. | Bachelor of Medicine/Surgery | “Pre-med” courses |
| Year -3 | General Diploma in Medical Science. | Bachelor of Medicine/Surgery | Doctor of Medicine degree |
| Year -2 | Diploma of Advanced Training in Medical Science | Bachelor of Medicine/Surgery | Doctor of Medicine degree |
| Year -1 | Diploma of Advanced Training in Medical Science | Bachelor of Medicine/Surgery | Doctor of Medicine degree |
| Year 0 | Diploma of Advanced Training in Medical Science | Bachelor of Medicine/Surgery | Doctor of Medicine degree |
| Year 1 | Resident specialist | Foundation doctor | First-year resident |
| Year 2 | Resident specialist | Foundation doctor | Junior resident |
| Year 3 | Resident specialist | Specialist registrar (min. 6y) | Senior resident |
| Year 4 | Resident specialist | Specialist registrar | Chief resident (min. 3 y, max 7 y) |
| Year 5 | Resident specialist | Specialist registrar | Chief resident |
| Year 6 | Chief assistant specialist (min. 2 y) | Specialist registrar | Chief resident |
| Year 7 | Chief assistant specialist | Specialist registrar | Chief resident |
| Year 8 | Consultant^*^ | Specialist registrar | Fellow or Attending physician |
| Year 9 | Consultant | Consultant^**^ | Fellow or Attending physician |
| Year 10 | Consultant | Consultant | Attending physician |

* After a minimum of 7 years of training

** After a minimum of 8 years of training
