# Supplementary material for: Modalities and Determinants of Career Paths in Pediatrics: A Survey of Former Pediatric Residents From Lille University Medical Center
Source: Front Pediatr. 2021 Nov 22;9:715269. doi: 10.3389/fped.2021.715269 (PMC8645605; doi:10.3389/fped.2021.715269)
Supplement: Supplementary file 2 [file Data_Sheet_1.docx]

**Supplementary Figure 1:** Retention of former Lille University Medical Center pediatric residents in Nord Pas-de-Calais (censored 10 years after the end of the residency) whose initial medical education was received in Lille (blue curve) or elsewhere Lille (red curve: Lille as a first choice for residency; green curve: Lille not the first choice) (p<0.001).

Supplementary Figure 2: Retention of former Lille University Medical Center pediatric residents in Nord Pas-de-Calais (NPC) (censored 10 years after the end of the residency) according to whether they had been a chief assistant specialist in NPC (red curve) or not (blue curve) (p<0.001).

**Supplementary Figure 3:** Retention of former Lille University Medical Center pediatric residents in hospital-based practice 10 years after the end of the residency according to start of residency (before 2003 (in blue) or since 2003 (in red)) (p=0.06).
