# Supplementary figures and images for: Modalities and Determinants of Career Paths in Pediatrics: A Survey of Former Pediatric Residents From Lille University Medical Center
Source: Front Pediatr. 2021 Nov 22;9:715269. doi: 10.3389/fped.2021.715269 (PMC8645605; doi:10.3389/fped.2021.715269)

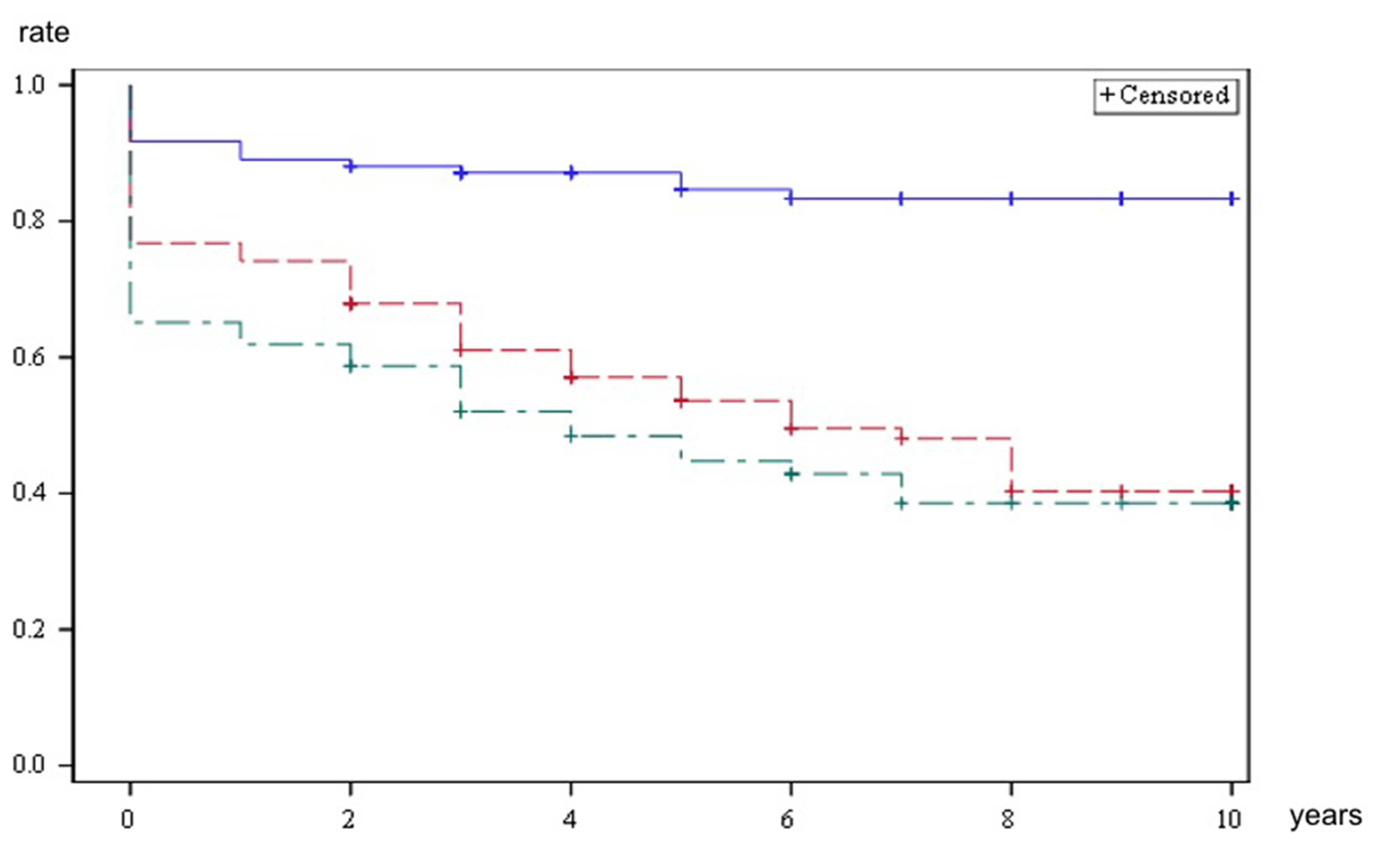

Supplement: Supplementary file 3 [file Image_1.jpg]

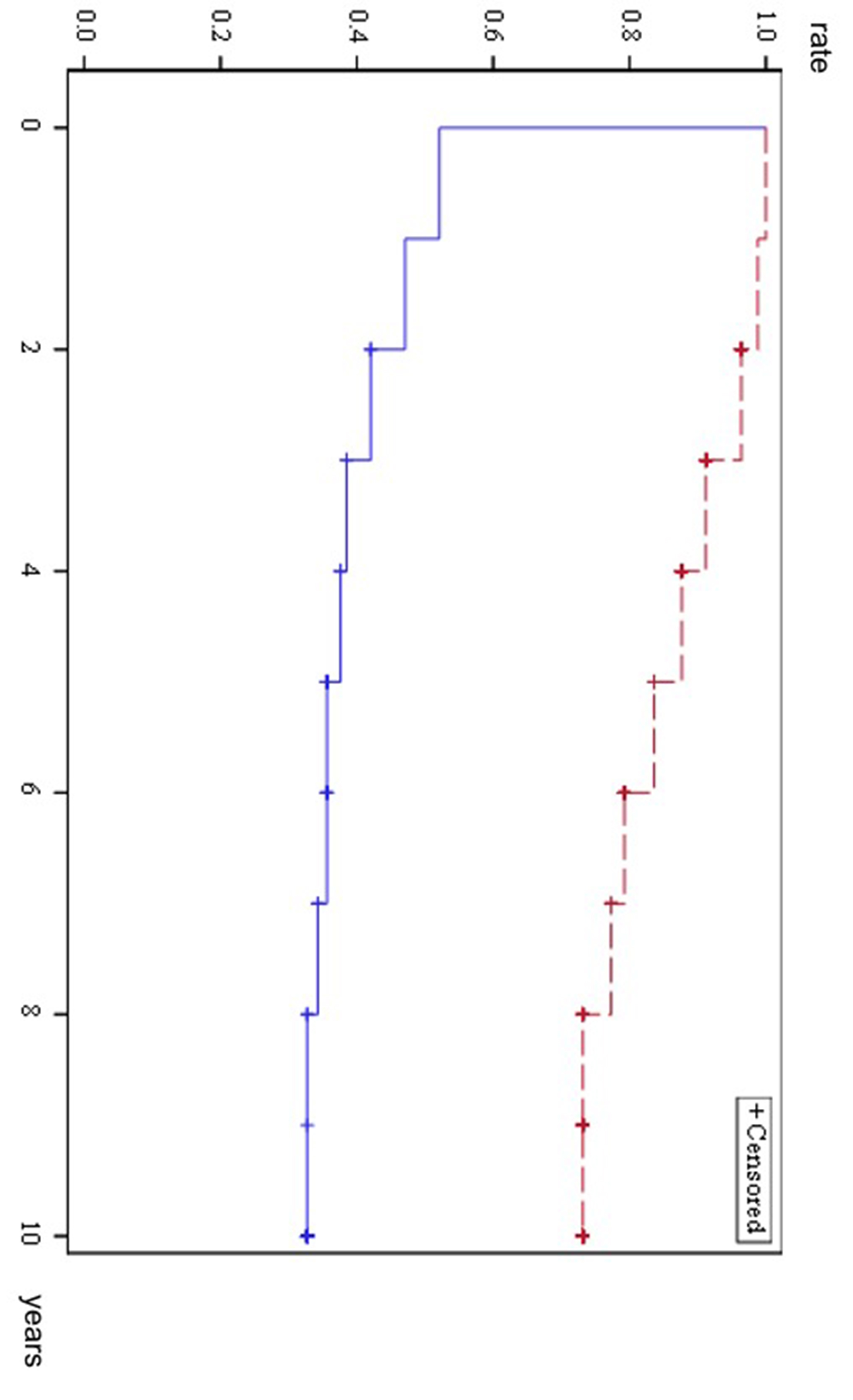

Supplement: Supplementary file 4 [file Image_2.jpg]

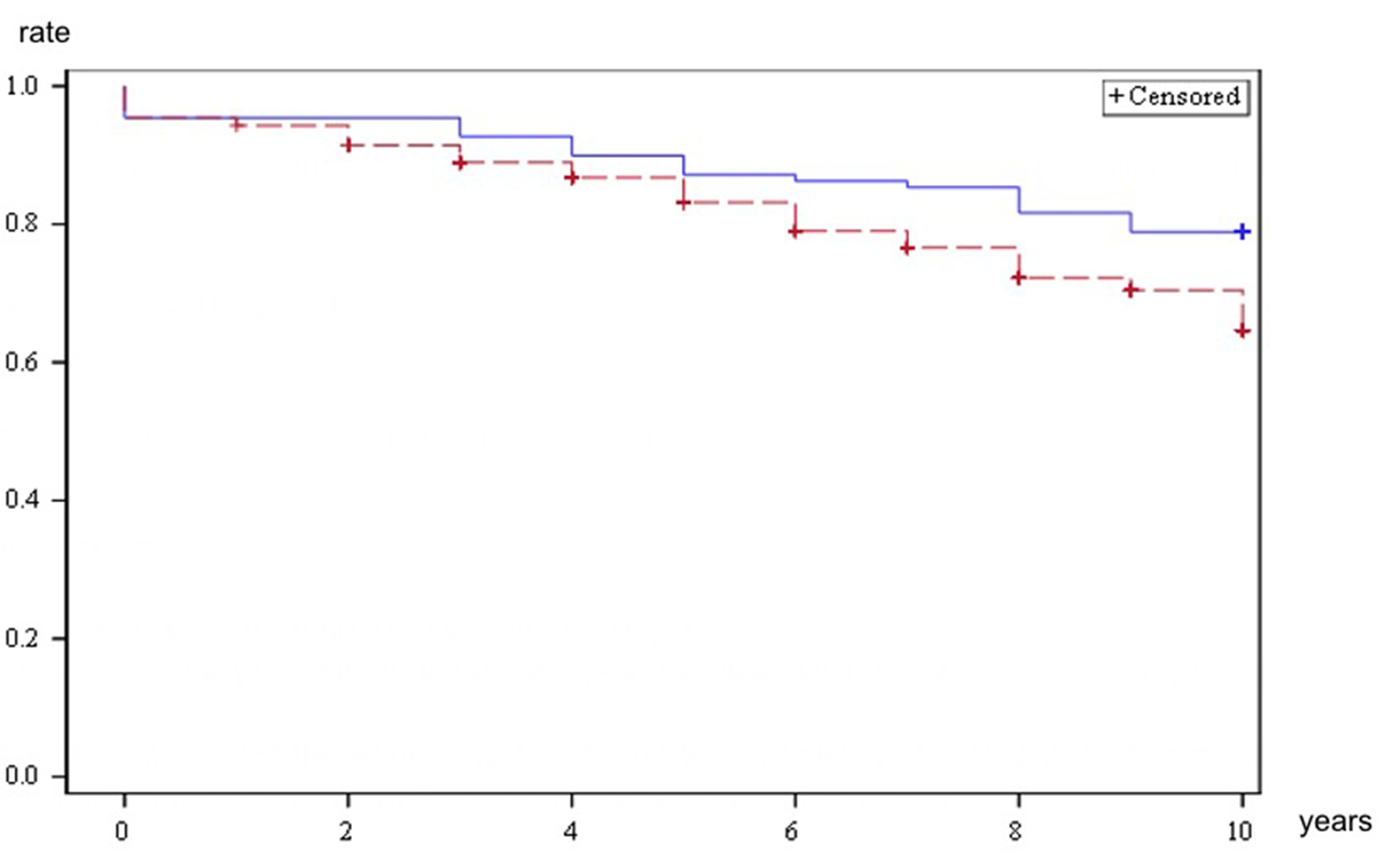

Supplement: Supplementary file 5 [file Image_3.jpg]
